# Bilateral carotid web successfully treated with endarterectomy, a case report

**DOI:** 10.1016/j.ijscr.2025.112120

**Published:** 2025-10-27

**Authors:** Mohammed A. Sadig, Abdullah G. Alsahwan, Omar Alothman, Alwaleed S. Althobaiti, Fahad Ahmed Z. Alshammari, Faris Alomran

**Affiliations:** a King Faisal Specialist Hospital and Research Center, Riyadh, Saudi Arabia; b Department of General Surgery, Faculty of Medicine, University of Dongola, Dongola, Sudan; c Department of General Surgery, King Fahd Hospital of the University, Imam Abdulrahman Bin Faisal University, Al-Khobar, Saudi Arabia

**Keywords:** Carotid web, Carotid endarterectomy, Carotid stenting

## Abstract

**Introduction:**

A carotid web is an uncommon vascular anomaly that has been identified as an important cause of ischemic stroke in young patients with no traditional atherosclerosis risk factors. This fibrous membrane present in the internal carotid artery tends to cause turbulent flow and thrombosis. We report a case of bilateral carotid web successfully treated by carotid endarterectomy.

**Case presentation:**

A 36-year-old female patient with a past medical history of non-epileptic seizures due to psychogenic factors and benign paroxysmal positional vertigo was presented to the emergency room with complaints of jerky movements of the right side of the body and then generalized jerky movements for 40 min. Neurological examination was unremarkable; the NIHSS score was 0. The CT scan showed an acute infarction in the right frontoinsular region. Additional imaging revealed a tiny linear defect within bilateral ICAs. Bilateral carotid webs were diagnosed upon cerebral angiogram, and the patient was diagnosed with cryptogenic stroke. She underwent right carotid endarterectomy. Postoperative recovery was uneventful, and the patient was discharged on the 3rd postoperative day. A subsequent left carotid endarterectomy was performed without complications.

**Discussion:**

Carotid webs play a key role in ischemic strokes, especially among the young patient population. Most are asymptomatic, but some patients have symptoms of TIA or stroke. Current management options include antiplatelet therapy and surgical intervention.

**Conclusion:**

This case highlights the importance of recognizing a carotid web as a potential cause of ischemic stroke and suggests that surgical intervention is warranted in symptomatic patients to prevent recurrent strokes. Further research is needed to optimize management strategies for this condition.

## Highlights

• A carotid web is a fibrous membrane in the internal carotid artery that can cause blood flow turbulence leading to ischemic stroke.
• Diagnosis relies on advanced imaging like CT angiography and cerebral angiogram, which reveal characteristic web-like lesions and turbulent flow.
• Medical management with antiplatelet therapy alone may not prevent stroke recurrence effectively in symptomatic patients.
• At present, there is no consensus regarding the most effective treatment option, whether it be carotid endarterectomy or stenting.

## 1 Introduction

A carotid web is a vascular entity that has been described as a common cause of ischemic stroke in younger populations with no atherosclerosis risk factors [1]. It is a fibrous membrane located in the lumen of the internal carotid artery (ICA), which can cause blood flow turbulence and lead to thrombosis. A carotid web is commonly asymptomatic, but it may cause an ischemic stroke. We present a case of bilateral carotid web that was successfully treated with carotid endarterectomy.

This case report has been reported in line with SCARE guideline [12].

## 2 Case presentation

**Fig. 1:**
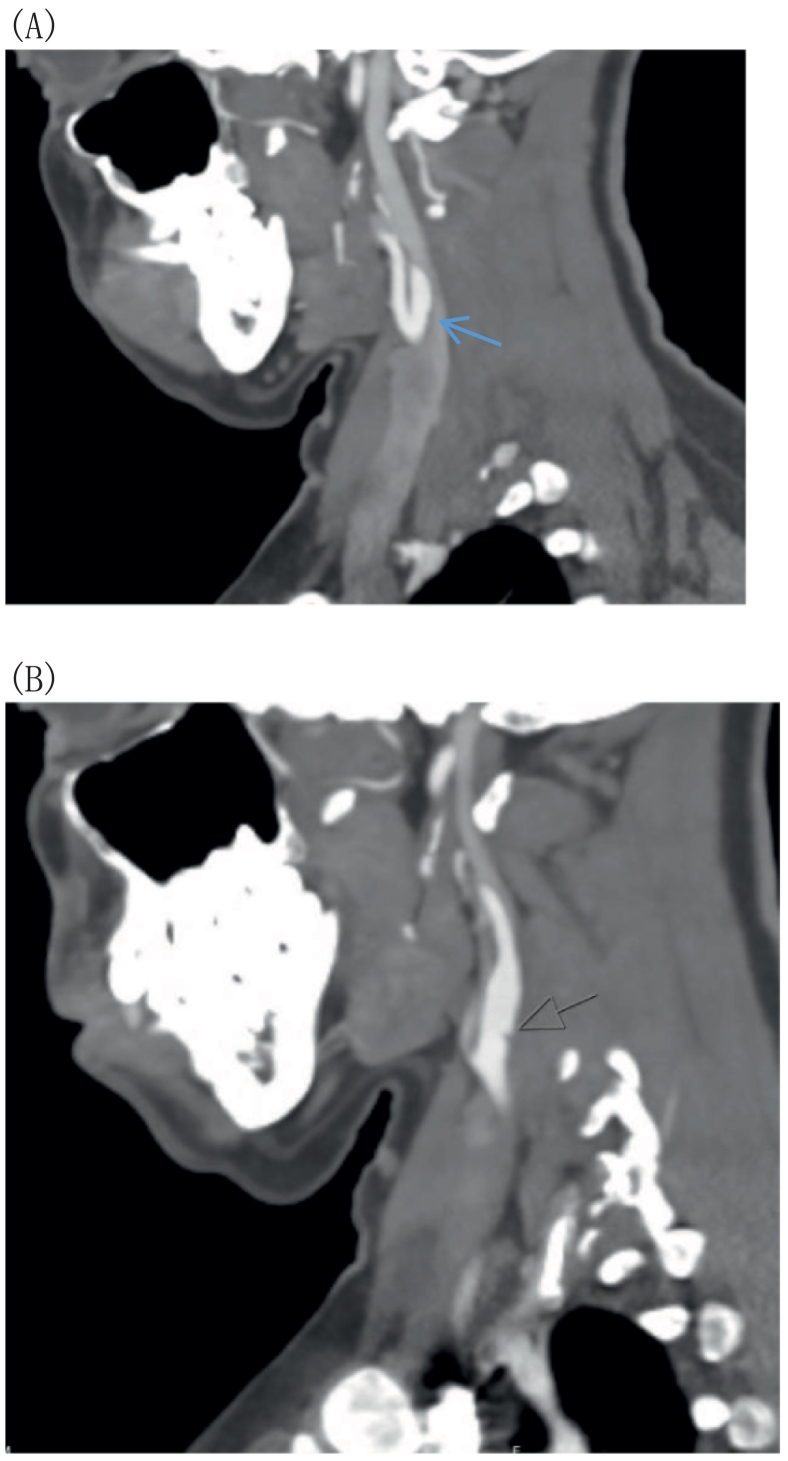
A and B: The sagittal view CT angiogram of the neck showed a shelf-like filling defect in the proximal internal carotid artery, with a blue arrow indicating the right ICA and a black arrow indicating the left ICA. (For interpretation of the references to colour in this figure legend, the reader is referred to the web version of this article.)

A 36-year-old female with a history of psychogenic non-epileptic seizures and benign paroxysmal positional vertigo was referred to the emergency department after she developed an episode of right-sided jerky body movements for 40 min, followed by generalized jerky body movements and forceful eye closure. There was no history of loss of consciousness, tongue biting, or urinary or fecal incontinence. The patient is a non-smoker and does not have a medical history of diabetes mellitus, dyslipidemia, or hypertension. Her NIHSS score was 0 at arrival. Her vital signs were unremarkable. Neurological examination showed normal findings in sensory, motor, deep tendon reflexes, and the Babinski sign. Brain CT showed an acute infarct in the right frontoinsular region in the territory of the right middle cerebral artery (MCA) without hemorrhagic transformation. Stroke code was called, and she had a brain CT (angiogram, perfusion studies), which showed no LVO, penumbra, or mismatch. Neck CT angiography demonstrated a small linear filling defect in the posterior wall of bilateral ICAs (Fig. 1.A and B).

**Fig. 2:**
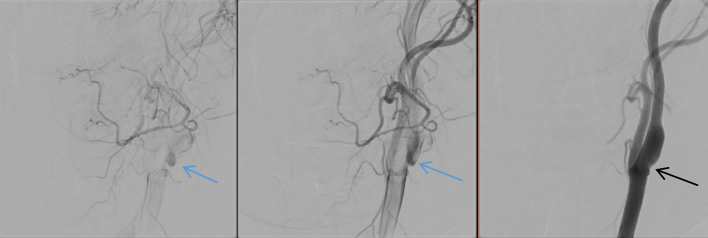
A digital subtraction cerebral angiography showed a filling defect in the posterior wall of the carotid bulb (black arrow). Then, there was stagnation of the contrast in the carotid web (blue arrows). (For interpretation of the references to colour in this figure legend, the reader is referred to the web version of this article.)

**Fig. 3:**
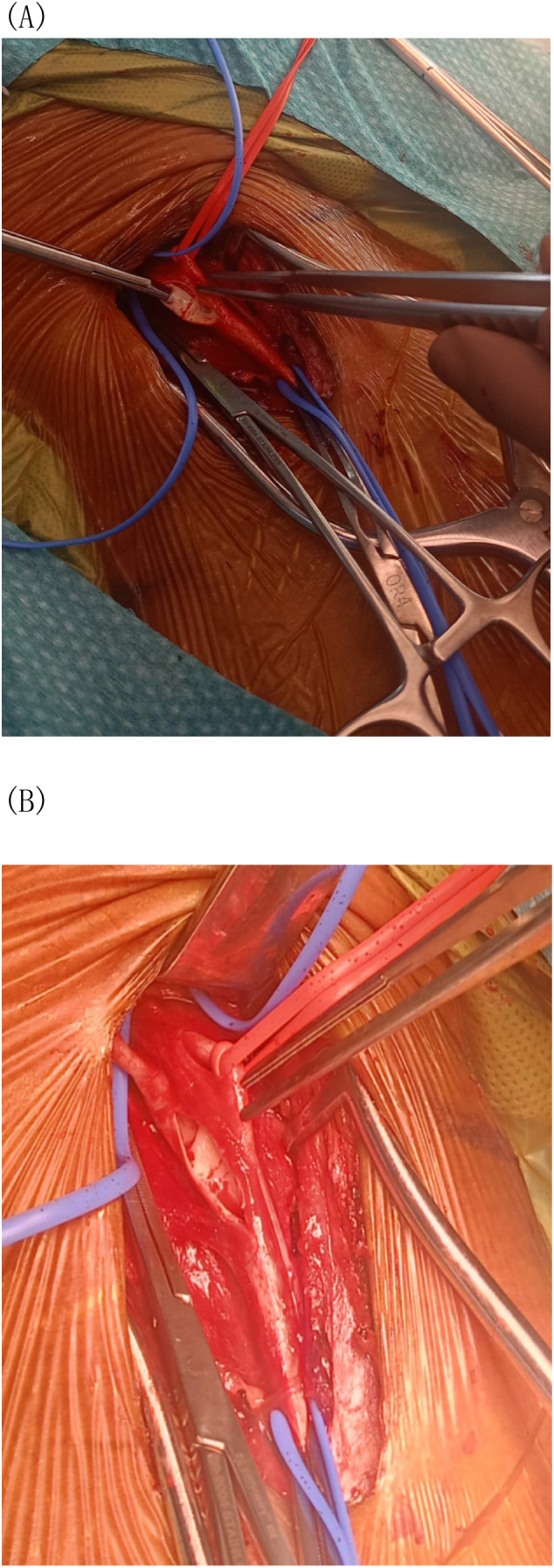
A and B: Intraoperative picture of ICA carotid web.

The patient was admitted for stroke work-up and was maintained on Aspirin 81 mg PO OD, Clopidogrel 75 mg PO OD, and Atorvastatin 40 mg PO OD. A cerebral angiogram demonstrated bilateral carotid webs with associated mural irregularities at the level of the carotid bulb, as well as turbulent flow with stasis of contrast at the carotid bulbs, but no angiographic stigmata of fibromuscular dysplasia (FMD) were noted (Fig. 2). Echocardiogram, 24-h Holter, and electrocardiography were obtained, and atrial fibrillation was excluded. Workup for thrombophilia and vasculitis was unremarkable. Family history was unremarkable. After ruling out all other causes of stroke, a diagnosis of cryptogenic stroke secondary to a carotid web was made. Right carotid endarterectomy was the consensus agreed upon by the multidisciplinary team. The patient had an uneventful right carotid endarterectomy. After the common, internal, and external carotid arteries were exposed, the vessels were clamped, and a long arteriotomy was made and extended to the internal carotid artery. The findings were a web-like structure at the posterior wall of the ICA (Fig. 3.A and B). The web was bluntly dissected and excised of the posterior wall using a Freer dissector, after which the arteriotomy was closed with a bovine pericardial patch and 6-0 Prolene. The postoperative course was uneventful, kept on a single antiplatelet and the patient was discharged home on postoperative day 3. Histopathology showed vascular tissue with myxoid changes and organization. Six weeks later, the patient returned for a left carotid endarterectomy with an uneventful postoperative period. Currently, she is being followed in our outpatient clinic without any active complaints, on a single antiplatelet for six months after surgery.

## 3 Discussion

A carotid web is a rare vascular pathology. This entity has been linked to severe adverse events and plays a prominent role as a cause of ischemic strokes, especially in younger patients and those lacking typical risk factors for atherosclerotic disease [1].

### 3.1 Pathophysiology

The pathophysiology of carotid webs is still not entirely understood [2]. It is believed to be due to hyperplasia of fibrous tissue in the internal carotid artery, which results in the formation of web-like structures at the posterior wall of the internal carotid artery. Micro-intimal dissections have been suggested in some studies as a potential cause of the web, but these webs are believed to be congenital [3]. This condition leads to blood flow turbulence and formation of thrombi that may be embolized into the cerebral circulation and may lead to ischemic stroke [4].

### 3.2 Clinical presentation

Most individuals with carotid webs are asymptomatic [5]. However, they may experience transient ischemic attacks (TIAs) or stroke. The manifestations usually include unilateral weakness, drooping of the face, or difficulties with speech, based on the affected cerebral hemisphere [5].

### 3.3 Diagnostic imaging

Carotid webs are usually diagnosed with advanced imaging, including noninvasive techniques (CT angiography or MRI/MRA). These are helpful for evaluating the web characteristics, dimensions, location, and any concomitant pathology. Some of these patients will require conventional angiography for diagnosis and treatment planning [6].

### 3.4 Management

To date, there is no strong level of evidence for the treatment of the carotid web. Treatment options include single or dual antiplatelet therapy, carotid endarterectomy, or stenting [7]. The guideline from the American Stroke Association recommends antiplatelet therapy for asymptomatic patients and those with a single event of ischemic stroke from the carotid web, but this recommendation is of a low level of evidence. For patients who have had multiple ischemic events, they recommend carotid intervention [8]. In the largest series of carotid webs, at a mean follow-up of two years, the rate of recurrent stroke was 0 % with surgical intervention (0 of 7 patients) compared to 30 % (6 of 20 patients) with antiplatelet therapy [9]. The 2023 ESVS guidelines propose that if no other source can be found following an extensive neurovascular workup in a symptomatic patient with a carotid web, they recommend that carotid endarterectomy or carotid artery stenting would be warranted to prevent recurrent stroke [10]. When comparing the optimal therapy between carotid endarterectomy and stenting, there is no consensus yet, as each carries a low risk of periprocedural complications—0.5 % compared to the complication risk of carotid intervention for atherosclerosis, which is ∼4 % [11]. Carotid stenting is a less invasive procedure; however, it necessitates long-term surveillance for in-stent stenosis, and the specifics of the disease remain unclear due to a lack of pathological data. Conversely, the benefits of carotid endarterectomy include the ability to establish a definitive pathological diagnosis.

## 4 Conclusion

This case highlights the need to identify a carotid web as a possible contributor to ischemic stroke among younger individuals with no atherosclerosis risk factors. In symptomatic cases, surgical interventions such as carotid endarterectomy or the placement of an endovascular stent may be required to mitigate the likelihood of subsequent strokes. Further studies are essential to develop effective management approaches for this condition.

## Author contribution

Mohammed Sadig: Principal investigator and writing.

Abdullah G. Alsahwan: Designs, writing and review.

Omar Alothman: Literature review, and data collection.

Alwaleed S. Althobaiti: Literature review, and data collection.

Fahad Ahmed Z Alshammari: Literature review, and data collection.

Faris Alomran: Overall supervision.

## Consent

Written informed consent was obtained from the patient for publication of this case report and accompanying images. A copy of the written consent is available for review by the Editor-in-Chief of this journal on request.

## Ethical approval

King Faisal Specialist Hospital and Research Centre waived the need for IRB approval due to the absence of patient identification in the study's enrolled participant.

## Guarantor

Dr. Abdullah G. Alsahwan.

## Research registration number

Not applicable.

## Provenance and peer review

Not commissioned, externally peer-review.

## Funding

Not applicable.

## Conflict of interest statement

The authors have no competing interests.
